# How Australian Health Care Services Adapted to Telehealth During the COVID-19 Pandemic: A Survey of Telehealth Professionals

**DOI:** 10.3389/fpubh.2021.648009

**Published:** 2021-02-26

**Authors:** Alan Taylor, Liam J. Caffery, Hailay Abrha Gesesew, Alice King, Abdel-rahman Bassal, Kim Ford, Jane Kealey, Anthony Maeder, Michelle McGuirk, Donna Parkes, Paul R. Ward

**Affiliations:** ^1^College of Medicine and Public Health, Flinders University, Adelaide, SA, Australia; ^2^Centre for Online Health, Centre for Health Services Research, The University of Queensland, Brisbane, QLD, Australia; ^3^Department of Epidemiology, School of Health Sciences, Mekelle University, Mekelle, Ethiopia; ^4^Barwon South West Telehealth Program, Barwon Health, Geelong, VIC, Australia; ^5^Digital Telehealth Network, South Australia Health, Adelaide, SA, Australia; ^6^Telehealth Tasmania, Tasmanian Health Service, Hobart, TAS, Australia; ^7^Northeast Health, Wangaratta, VIC, Australia; ^8^Flinders Digital Health Research Centre, Flinders University, Adelaide, SA, Australia; ^9^Menzies School of Health Research, Darwin, NT, Australia; ^10^Agency for Clinical Innovation, New South Wales Health, St Leonards, NSW, Australia

**Keywords:** Australia, telehealth, COVID-19, survey, mechanisms, realist

## Abstract

**Background:** In Australia, telehealth services were used as an alternative method of health care delivery during the COVID-19 pandemic. Through a realist analysis of a survey of health professionals, we have sought to identify the underlying mechanisms that have assisted Australian health services adapt to the physical separation between clinicians and patients.

**Methods:** Using a critical realist ontology and epistemology, we undertook an online survey of health professionals subscribing to the Australian Telehealth Society newsletter. The survey had close- and open-ended questions, constructed to identify contextual changes in the operating environment for telehealth services, and assess the mechanisms which had contributed to these changes. We applied descriptive and McNemar's Chi-square analysis for the close-ended component of the survey, and a reflexive thematic analysis approach for the open-ended questions which were framed within the activity based funding system which had previously limited telehealth services to regional Australia.

**Results:** Of the 91 respondents most (73%) reported a higher volume of telephone-based care since COVID and an increase in use of video consultations (60% of respondents). Respondents felt that the move to provide care using telehealth services had been a “forced adoption” where clinicians began to use telehealth services (often for the first time) to maintain health care. Respondents noted significant changes in managerial and medical culture which supported the legitimisation of telehealth services as a mode of access to care. The support of leaders and the use personal and organisational networks to facilitate the operation of telehealth service were felt to be particularly valuable. Access to, and reliability of, the technology were considered extremely important for services. Respondents also welcomed the increased availability of more human and financial resources.

**Conclusions:** During the pandemic, mechanisms that legitimise practise, build confidence, support relationships and supply resources have fostered the use of telehealth. This ongoing interaction between telehealth services, contexts and mechanisms is complex. The adoption of telehealth access to enable physically separated care, may mark a “new context;” or it could be that once the pandemic passes, previous policies and practises will re-assert themselves and curb support for telehealth-enabled care.

## Introduction

During the COVID-19 pandemic physical separation between clinicians and patients was encouraged to help reduce the risk of community transmission of the virus (1). To achieve this separation telehealth services were used as an alternative method of health care delivery. This afforded protection to both patients and health care providers (2). A telehealth service is defined by the International Organization for Standardization as “healthcare activity undertaken using information and communications technologies to deliver healthcare and transmit health information over both long and short distances” (3). Telehealth may use synchronous communications technologies such as the telephone or video conferencing or asynchronous technologies such as web-based communications, messaging and monitoring.

Australia had well established telehealth services before the pandemic. Canada, New Zealand and the USA were similarly positioned. National and regional governments in these countries were able to rapidly adjust regulations and payments (4, 5). These changes resulted in a growth in the use of telehealth as demonstrated by, virtual consultations grew from 1,800 each week to 19,000 a week in British Columbia, Canada (6); and in New Zealand telehealth consultations rose ten-fold to 34,500 per week (7), although this figure has since declined.

Substantial increases in the proportion of consumers using telehealth consultations in the USA have been reported (8). American Well, a corporate telehealth service, has stated that 80% of its providers now provide care using telehealth services compared with 20% previously and patient use of telehealth services has increased by a factor of 9 times (9). In France teleconsultations have increased to 11% of all consultations where any application can be used to conduct teleconsultations, including consumer applications such as Skype, WhatsApp, and Facetime. Also in France, tele-monitoring of COVID-19 patients can be performed by nurses and is 100% reimbursable (10).

The introduction of temporary government subsidies radically expanded Australians' access to telehealth under Medicare; and telehealth access to many services was funded by Australian State and Federal governments (11). As a result, there was a substantial increase in the use of telehealth during COVID-19, in particular during stricter lockdowns.

We sought to understand how Australian health services have adapted to the use of telehealth during the pandemic. This research had two broad aims. Firstly, to determine the extent and type of changes that have occurred to telehealth services in Australia since the start of the COVID-19 pandemic, and secondly to probe for explanations as to why these changes occurred.

## Materials and Methods

### The Australian Health Care Context

The Australian health care system is generally regarded as providing high quality, affordable health care services. Australia's health care system and funding models are a complex blend of private and public services. Total Australian health expenditure as a percentage of GDP was 10.3% in 2016 (12). Australia's universal health care system is known as the Medicare Benefits Scheme (MBS). The MBS is funded by a Medicare levy which is 2% levy on taxable income for people earning above a threshold salary. MBS subsidises medical services provided by both General Practitioners (GP) and specialists, as well as a very limited number of allied health services. Many medical practitioners also charge the patient a gap fee which is additional out-of-pocket cost for the patient. Since the commencement of the MBS, the failure to index MBS subsidies has resulted in rising medical fees leaving patients with a larger out-of-pocket gap payment (13).

Primary care services are predominantly provided by privately practising general practitioners (GPs). GP consultations are subsidised using a time and complexity-based fee structure by MBS. In areas where a private model is unsustainable (e.g., remote communities) state health departments or non-government organisations (e.g., the Royal Flying Doctors Service) may provide GP services using salaried doctors.

Acute care services are owned and managed by State and Territory governments with funding coming from State or Territory Governments and the Commonwealth Government. This is often called the public hospital system. Acute hospitals are funded under an activity-based funding (ABF) model. However, hospitals that are not viable under an ABF model are block funded. Public hospitals provide the majority of emergency departments that operate in Australia.

Many Australians also carry private health insurance. Private health insurance predominantly covers private hospital admissions, private dental services and private allied health services. The Australian government provides a 30% rebate on private health insurance premiums to Australians below an earning threshold. Further, the Australian Government has introduced tax penalties for people over 30 years of age who do not carry health insurance. Despite incentives there is a declining number of Australians with private health insurance. Private health insurance membership for hospital admission has fallen from 50% in 1984 to 47.4% in 2015 and 46.5% in 2017 (14, 15).

### Telehealth in Australia

Australia is a large country with intensively settled areas in coastal regions and sparse populations in non-coastal areas. Uneven distribution of the health workforce, particularly specialists, is associated with differential access to health services and facilities for the general population. Regional residents tend to fall into lower income brackets, so the cost of healthcare and travel becomes important. Australian telehealth service models attempt to reduce patient travel to specialist centres by enabling care to be provided into patients' homes or diverting patients to local regional facilities, which then support remote consultations.

In Australia, telehealth services are largely operated by federal and state governments although there is a growing private sector. The federal Government funds telehealth services, under the MBS. States fund teleconsultations within their public hospital systems using ABF. Some states such as Queensland and South Australia run internal video consultation networks which are now gradually opening up to use by primary care practitioners. The Queensland system is by far the largest in terms of usage and has run on an internal network since 2001.

Prior to the COVID-19 pandemic the Australian Federal Government supported a limited range of payments to specialists for video-based consultations as part of the MBS. Between 2012 and 2019 telehealth MBS items were subject to only minor adjustments, but use of these items has steadily increased reaching about 230,000 consultations during the 2018–2019 financial year. State-based public hospitals also provide significant numbers of video-based consultations, for instance Queensland Health provided over 100,000 consultations in 2018. Nevertheless, depending on the speciality, video consultations in both Queensland and Australia as a whole, represented <1% of all consultations prior to the pandemic (16, 17).

Telehealth services grew out of the need to support regional health professionals. Educational use initially dominated (18), but, proportionally, has declined as clinical use has increased. In Australia, telehealth services are almost synonymous with video conferencing consultations (video consultations) between hospital-based specialists and patients in regional areas, at home or supported by local general practitioners or rural clinics. Telephone-based services are focused on providing advice and care directly to patients. Asynchronous telehealth services have been slow to develop or exist under the banner of “eHealth” services, providing diagnostic information between clinicians.

Electronic health records are available across public and private hospitals. In 2018, a survey of Australian general practitioners found that 87% are completely digital and maintain no paper records (19). Telehealth services and eHealth share a dependence on evolving information and communication technologies (ICT). However, eHealth has in the main focused on improving the level of automation and access to information in healthcare, while telehealth services are largely concerned with improving access to care.

Telehealth services rely on communications technologies. In 2007, a new Australian government promised to build the National Broadband Network (NBN) based on an optical fibre telecommunications network. The NBN provides broadband access to 93% of the Australian population, with rural areas obtaining access through fixed wireless and satellite.

### Australian Responses to COVID-19 Pandemic

In Australia, the Commonwealth government enabled a wide range of medical professionals to claim rebates using the MBS for consultations that had not previously been eligible for telehealth access. As in-person consultations declined regulations and funding packages were developed to improve the capacity of health services to talk to or see patients remotely using ICT.

Telehealth consultations (telephone and video) formed 28% of all federally funded consultations. Primary care (by GPs), specialist and mental health consultations were the most used. Video conferencing comprised 8% of federally funded telehealth consultations. Specialist consultations made greater use of video conferencing. Mental health consultations, for which video conferencing is an established modality, were provided in almost equal proportions using the telephone and video conferencing (20).

An Australian Bureau of Statistics (ABS) survey (21) reported that “in November (2020), almost one in six (18%) Australians used a telehealth service in the previous four weeks. This was similar to the use of a telehealth in June (20%) and May (17%).” According to the same survey “almost half (49%) reported they were likely to use telehealth services in the future.” A separate national study of people's experiences and satisfaction with telehealth during the COVID-19 pandemic in Australia by Isautier et al. (22) found that “telehealth appointments were reported to be comparable to traditional in-person medical appointments by most of our sample (p. 2).”

### Study Design and Population

We undertook an online cross-sectional survey of subscribers to the Australian Telehealth Society (ATHS) newsletter between July 5th, 2020 and September 10th, 2020. The Flinders University Social and Behavioural Research Ethics Committee approved this research (Project number 8668). The participant population was chosen according to the recommendation by Manzano (23) because it was likely to reach practitioners of telehealth services who “have specific ideas on what it is within the programme that works (mechanisms) because they are likely to have broad experience of successes and failures, and some awareness of people and places for whom and in which context the programme works” (p. 8). The survey therefore sought to elicit informed views of telehealth practitioners and was not designed to seek the opinions of a broad section of the Australian healthcare community. The survey was administered using a Flinders University Qualtrics software licence that enables respondents to complete the survey online anonymously or via a link contained in an emailed invitation and provides descriptive statistical analysis of the results.

### Theoretical Framework

The theoretical approach of this study is founded in a critical realist ontology and epistemology, which views reality as stratified into different levels of activity and observability, and looks for explanations of changes in reality in the form of generative causal mechanisms (24). Recent research into telehealth services has found that continued operation, development, or sustainability of telehealth is contingent on and sustained by interactions between contexts and telehealth services through four key mechanisms, which:

- legitimise practise based on explicit and implicit sociotechnical codes including strategies, guidelines, and clinical routines;
- build confidence through accepting technology, management of the risks, and creation of trust in practise;
- build relationships between stakeholders; and
- acquire resources, such as information and communications technology, human resources, and funding [(25), unpublished doctoral thesis, Flinders University].

Contexts have been identified as crucial to understanding the operation of health services (26, 27). Contexts perform a dual role by hosting mechanisms and changing as a result of interactions with mechanisms. Organisational contexts host the norms, processes, and practises of telehealth services and professional contexts reflect established clinical practises, culture authority, and roles.

### Tools and Measurement

The survey has close- and open-ended questions and was constructed to identify changes that had occurred in the contexts within which telehealth services operate, and assess the mechanisms which had contributed to these changes. A total of 40 survey questions were formulated. Because previous work has shown that socio-cultural elements have a far stronger influence on telemedicine adoption and effectiveness than choice of a specific technology solution (27), questions regarding the type of technologies used by telehealth services were not included in this survey.

Questions related to organisational and professional contexts, postulated mechanisms, changes in services, patient experiences and acceptance of telehealth services, sought to understand what constraints on organisational deployment of telehealth services exist. Other questions probed the interaction between professional cultures and the operation of telehealth services? For example:

- Has telehealth been legitimised by clinicians, management and technologists in your organisation?
- How has confidence been built in telehealth services?
- How have professional relationships been maintained?
- What sorts of resources have been important operating telehealth services since the beginning of the pandemic?
- What sorts of changes supported the increased acceptance of telehealth services?
- How has the modality, scope, volume and quality of healthcare delivery using telehealth services changed?
- What changes have there been in the patient experience?
- Have the needs of vulnerable populations been considered?
- Is the provision of remote consultations by your organisation or unit now routine?

The survey was designed to elucidate responses to each research question and encouraged free text comments by respondents on each topic. Respondents were asked to rate the relative importance of proposition or possible factor using five-point sliding Likert items and free text comments.

### Data Analysis

For the quantitative (close-ended) component, data were exported from Qualtrics to Excel and then to IBM SPSS for analysis (28). We have applied descriptive and inferential statistics. Proportion and percentages were calculated to describe the main variables of the study. While most data were on a five-point scale (1–5), we have dichotomized the results in to two categories (below and above 3, the neutral) for inferential analysis (29). We applied McNemar's Chi-square analysis, assuming “all categories (expected probabilities) have equal probability” to assess the relationship between selected variables. We have also calculated the Overall Cronbach's Alpha and Maximum Cronbach's Alpha when an item deleted for each item.

For the qualitative component, analysis of respondent comments to each open-ended question was supported by manual methods and NVivo qualitative analysis software (30). Analysis of the free text comments applied a reflexive thematic analysis approach (31) by two independent coders to find repeated meanings. Initially, one coder generated initial themes by identifying interesting features of the data. The second coder used an initial theme set of organisational and professional contexts, legitimisation of practise, building confidence and relationships and acquisition of resources which were aligned with previous findings. The two coders then combined their results and collaborated on their interpretation by iteratively reflecting on and refining themes over a period of several weeks.

## Results

Ninety-one (*N* = 91) participants across Australia responded to e-mail (*n* = 65) and anonymous (web link) (*n* = 26) invitations. The majority of the participants were from the Australian states of Victoria (*n* = 27) and Queensland (*n* = 25) and New South Wales (*n* = 13). In total, 54 (59%) participants were directly involved in the provision of telehealth services compared to 34 (37%) participants who were involved indirectly. The role of about one-third participants who were directly involved in the provision of telehealth services was health service manager or researcher. Two-thirds of participants were practising health professionals in general practise, specialist medical, nursing or allied health roles. Of the 29 participants who involved in indirect telehealth services provision, most were technical support (*n* = 13), administrator (*n* = 7) and training or education (*n* = 5) (Table 1).

**Table 1:** Characteristics of participants.

**Characteristics of participants**		***n* (%)^A^**
Distribution Channel, *n* = 91	E-mail	65 (71.4)
	Anonymous	26 (28.6)
Q2.4 Work place, (*n* = 81)	Australian Capital Territory	2 (2.5)
	Queensland	25 (30.9)
	New South Wales	13 (16)
	Northern Territory	1 (1.2)
	South Australia	4 (4.9)
	Tasmania	1 (1.2)
	Victoria	27 (33.3)
	Western Australia	4 (4.9)
	Outside Australia	4 (4.9)
Q2.1 Level of involvement of telehealth provision, (*n* = 88)	Directly	54 (59.3)
	Indirectly	34 (37.4)
Q2.2. Role of direct involvement of telehealth provision, (*n* = 50)	Health service manager, coordinator, or researcher	18 (36)
	Others^B^	32 (64)
Q2.2. Role of direct involvement of telehealth provision, (*n* = 50)	General practise	4 (8)
	Specialist medical	11 (22)
	Nursing	6 (12)
	Allied health	11 (22)
	Health service manager	15 (30)
	Researcher	3 (6)
	Coordinator	–
Q2.3 Role of indirect involvement of telehealth provision, (*n* = 29)	Administrator	7 (24.1)
	Equipment supplier	1 (3.4)
	Services provider	3 (10.3)
	Technical support	13 (44.8)
	Training or education	5 (17.2)

A The percentage is “Valid percent.”

B *Others' refers to General practise, Specialist Medical, Nursing and Allied Health*.

We reported on how healthcare, organisations and professions have adapted to increase the proportion of care provided using telehealth services. We also explored respondent's views on the relative importance of legitimisation, confidence building, relationships and resources in enabling these changes.

### Changes in Healthcare Delivery

Respondents directly involved in provision of telehealth services were asked to rate their perceptions of changes to healthcare delivery since the start of the pandemic on a five-point Likert item (Table 2).

**Table 2:** Characteristics of telehealth resulting from COVID.

**Changes in healthcare delivery**	**Number of respondents in agreement with statement (*n*) (%)**	**Respondents (*n*)**
Higher telephone consultation volumes	27 (72.9)	37
Higher video consultation volumes	21 (60.0)	35
Much better patient satisfaction (telephone) reported	14 (51.9)	27
Much better patient satisfaction (video) reported	13 (54.1)	24
Time spent was about the same as face-to-face consultations	11 (35.5)	31
Main purpose was management or treatment of non-COVID-19 health conditions	19 (55.9)	34
Additional measures had probably or definitely been put in place to support vulnerable patient cohorts	21 (53.8)	39
Extending the type of services offered	30 (52.6)	57
Changing geographical criteria	16 (30.8)	52
Applying different funding or payment criteria	14 (31.8)	44
Some or strong agreement that telephone consultations were routine	28 (82.4)	34
Some or strong agreement video consultations were routine	31 (91.1)	34
No firm opinion existed on whether monitoring of conditions was routine	–	30

The majority of respondents reported increased consultation volumes. For example, one manager stated “*Our already established tele-rehabilitation program was able to rapidly increase activity from an average 600 service events per month to a peak of 3,300 in April.”*

For other services such as healthcare language interpreters, a complete change in the delivery modality occurred. Interpreter services came to rely on phone and video communications because of the risks of losing staff should they become ill. There was some or strong agreement between respondents that telehealth services were now considered as routine care with one respondent stating “*telehealth (videoconferencing) services is face-to-face services, as we are seeing the patients face and they are seeing ours - our patients receive the same care no matter where they are*.” Respondents also noted that the introduction of telehealth consultations had changed the workload for administrative staff because “*while the time taken for consultations is slightly less, the administrative time to arrange appointments has significantly increased, as well as the time required to ensure billing is compliant*.”

### Organisational Adaptation

Respondents were asked to comment on factors that they perceived influenced the acceptance of telehealth services. Governmental or organisational decisions (*n* = 23, *N* = 52), and the availability of payments (*n* = 20, *N* = 48) were cited most frequently as providing a great deal of support. Health reforms or strategies (*n* = 19, *N* = 48), inclusion of remote consultations in appointment systems (*n* = 22, *N* = 46) and remote consultations becoming part of daily routines (*n* = 21, *N* = 52) were felt to have provided a lot of support for the increased acceptance of telehealth services. A respondent noted that “*both clinicians and families have been ‘pleasantly surprised’ and significantly More buy in now*” while another felt that the changes had not been easy to make, with “*Clinicians forced to adopt - removed some behavioural barriers to uptake and encouraged perseverance until able to competently use telehealth platforms. Noting lot of frustration due to this requirement though*!”

### Professional Adaptation

Respondents were asked to compare the extent to which professional managerial, medical or technical cultures assisted the use of telehealth services before and after the outbreak of COVID-19. Application of McNemar's Chi-square test showed that managerial and medical cultures have significantly changed the extent to which they support telehealth (Table 3).

**Table 3:** Changes to workplace culture about telehealth resulting from COVID.

**Professional**	**Assisted in the**	**Most frequent**	**Below median,**	**Above median,**	**McNemar's Chi-square**
**Group**	**use of telehealth**	**response**	***n* (%)**	***n* (%)**	***p*-value**
Managerial culture	Before the outbreak of COVID-19	A moderate amount	17 (51.5)	16 (48.5)	0.001
	After the outbreak of COVID-19	A lot	5 (10.9)	41 (89.1)	
Medical culture	Before the outbreak of COVID-19	A moderate amount	17 (65.4)	9 (34.6)	0.001
	After the outbreak of COVID-19	A lot	5 (12.5)	35 (87.5)	
Technical culture	Before the outbreak of COVID-19	A lot	14 (50)	14 (50)	0.125
	After the outbreak of COVID-19	A lot	7 (20)	28 (80)	

Changes to culture were, in the view of several respondents, “enforced” as a result of a risk analysis that compared the risks of infection control during place-based, in-person care with the risks of physically separated care using telehealth services. According to one respondent:

*The external huge risk of COVID made inroads into the status quo - where change was necessary/mandated in order to offer continued care to clients. That is/was the opportunity in a nutshell- the nature of normal risk aversion and standard fear of change got beaten to death by the much larger imposed risk profile*.

Another respondent indicated the extent of change in attitudes that had occurred compared to the “*old fears* [which] *have, in many cases been proven to be baseless. It was always the case, but medical opinion is very challenging to impose change on*.”

### Legitimisation of Services

Respondents reported that while the legal and contractual arrangements influencing use of telehealth services had not changed during the pandemic, financial constraints had become slightly weaker (*n* = 12, *N* = 38) and collaboration with other organisational units (*n* = 28, *N* = 42), medical specialities or allied health (*n* = 29, *N* = 42) and information technology specialists (*n* = 19, *N* = 40) had all become a little easier. On the one hand the changes to the MBS items were welcomed “*We have been allowed to consult via phone to reduce patients coming into hospital during the COVID 19 time. Previously we were not allowed because this service could not be billed*.”

In Australia, at the beginning of the pandemic patients could arrange telehealth appointments with any GP. Following lobbying by some professional associations who felt that their members were losing business to new entrants to this sector, the government restricted funding for telehealth appointments to patients who had visited the GP practise within the previous 12 months. Consequently, the initial loosening of restrictions to enable all patients to be seen by telehealth, followed by a stipulation that only patients who had attended the same practise within the past 12 months could be seen remotely meant that for one provider:

*For my business, the pull-back of GP telehealth rebates, restricting eligible consultations to a patient's “usual” GP, caused my client base to dwindle overnight. A large proportion of my clients are in vulnerable rural and remote areas, and can't afford health services with no rebates*.

### Building Confidence in Practise

The survey explored the importance of influences in building confidence in using telehealth services (see Table 4).

**Table 4:** Factors associated with clinician confidence in telehealth.

**Perceived factors in building confidence**	**Number of respondents in agreeance with statement *n* (%)**	**Respondents**
It was extremely important to have easy to use systems	37 (67.2)	55
It was extremely important to know systems are private and secure	22 (40.7)	54
It was very important to get technical or administrative support quickly	24 (43.6)	55
It was very important to triaging the most suitable patients	22 (40.7)	54
It was moderately important to trust colleagues	21 (38.8)	54

The confidence of health professionals in telehealth practises was felt to be a key issue because according to one respondent “*clinicians do not want to look silly in front of their patients*.”

Respondents placed particular emphasis on having easy to use systems which are private, secure and well supported by administrative and technical staff. A respondent reported that “*Confidence has grown hugely. Most clinicians are now savvy and adaptable on any platform*.” The ability to choose the most suitable patients to receive care using telehealth services was also thought to be important. A respondent that had been using telehealth services for some time felt that the increased acceptability of telehealth services was not an overnight phenomenon because “*I have worked in reviewing patients via telehealth for over the last 4 years and have slowly watched an increase in acceptability and confidence in the ability to provide healthcare in this manner.”*

Our survey explored how easy has it has been to maintain professional relationships with colleagues at a distance using ICT since the beginning of the COVID-19 pandemic. Respondents reported that while email use had not changed it had become a little easier to use the telephone (*n* = 10, *N* = 24) and much easier to use video conferencing (*n* = 22, *N* = 39) for this purpose. Respondents also reported that achieving consensus with clinicians (*n* = 25, *N* = 44), management (*n* = 22, *N* = 43) and technologists (*n* = 21, *N* = 35) in their organisation on how to implement telehealth services had all become a little easier, with one respondent noting that clinical dominance of telehealth service provision now accommodated greater contributions from other members of the service team “*The team ethos has been reinforced with a much more equal attitude between team members ie a service philosophy rather than ‘clinical is king’*.”

### Supportive Relationships

When respondents were asked about the importance of factors in maintaining relationships that support the use of telehealth services for access to care since the beginning of the COVID-19 pandemic, good leadership, networks and teamwork were mentioned as extremely or very important. Whereas, communities of practise and formal partnerships were perceived as less important (Table 5).

**Table 5:** Relationship factors that support the use of telehealth.

**Perceived relationships factors that support the use of telehealth**	**Number of respondents in agreeance with statement *n* (%)**	**Number of respondents *n***
Having good leadership was extremely important	28 (59.5)	47
Personal and organisational networks were very important	27 (64.2)	42
Teamwork was very important	24 (60.0)	40
Communities of practise very important	15 (45.5)	33
Formal partnerships were very important	15 (51.7)	29

Comments from respondents indicated that while the factors listed in Table 5 were important, experiences varied. On the question of leadership one respondent felt that “*generally culture has changed around use of telehealth, now being promoted throughout the organization*,” while another complained that “*it has been difficult to get the ear of management as they are occupied with dealing with COVID*.”

### Resourcing Services

The non-financial resources which were perceived to be extremely or very important to operating telehealth services are listed in Table 6. However, when asked about whether ICT systems in their organisations were able to support telehealth services, respondents were a little hesitant and could only probably confirm that their systems could exchange information (*n* = 20, *N* = 41) and connect with different video conferencing systems (*n* = 20, *N* = 40). Nevertheless, they did believe that these systems were able to maintain patient privacy (*n* = 23, *N* = 44). Access to and reliability of the technology was most frequently considered extremely important.

**Table 6:** Non-financial resources needed for telehealth.

**Importance of resources to operating telehealth**	**Number of respondents in agreeance with statement *n* (%)**	**Number of respondents *n***
Access to suitable technology was extremely important	29 (56.9)	51
Reliable of technology was extremely important	34 (0.68)	50
Staff training was very important	21 (51.2)	41
Access to appropriate physical space was very important	17 (44.7)	38

Respondents welcomed the increased availability of resources, such as “*more personnel available to assist setting up telehealth, more equipment, changes to protocols to make telehealth easier.”* However, some respondents reported difficulty obtaining and supporting services because “*Australia quickly ran out of basic office equipment (webcams, iPads etc became harder to source) Technical support roles were stretched to support across a broader scope and assist with rapid uptake and training*.” Despite these reservations, most respondents indicated that somewhat more technical support for users, devices, communications (such as the internet), and training had been made available to them since the beginning of the COVID-19 pandemic (*n* = 15–20, *N* = 28–35). Respondents were also asked if their organisation uses a National Broadband (NBN) connexion. Of 53 respondents to this question 25 were able to confirm use of the NBN and 15 were unsure. Of those who were sure they used the NBN, 22 respondents indicated the NBN performed satisfactorily, well or extremely well.

## Discussion

In this survey 73% of 91 the respondents reported a higher volume of telephone-based consultations compared with 60% of respondents reporting increased uptake of video-based consultations. Telehealth services were used mainly for the management of non-COVID health conditions. Many respondents felt that the move to provide care using telehealth services had been a “forced adoption” where clinicians began to use telehealth services to provide care (often for the first time) and persevered until they felt comfortable with this modality of healthcare delivery. Most respondents identified a learning curve, but perseverance resulted in confidence to use telehealth. Respondents also perceived significant changes in managerial and medical culture, and the legitimisation of telehealth services as a mode of access to care, all of which were important in the uptake of telehealth. The finding that leadership, and personal and organisation networks were perceived as being more important than formal partnerships and communities of practise is supported by previous Australian studies (32). Access to, and reliability of the technology was considered extremely important. Respondents welcomed the increased availability of resources, more personnel available to assist setting up telehealth, more equipment, and changes to protocols to make telehealth easier. The lower use of video conferencing may be due to a variety of reasons (33), but in part may be explained by variations in need (for instance a video consultation may not be needed when renewing a prescription), and variations in the availability of cameras in consulting rooms or poor interoperability between video conferencing solutions (34).

One of the key contextual changes in Australia has been that the MBS, Australia' universal health system, and associated regulations have both legitimised and resourced the use of telehealth across a much greater range of healthcare activities than were previously allowable. Consequently, there have huge increases in the volume of telephone and video-based consultations between doctors and patients for services funded by the Australian Government via MBS. The new telehealth rebate items in the MBS mirrored the pre-exiting in-person consultation items by adding rebates for telehealth (video) and telephone consultations. In all, 279 COVID-19 items have been introduced (1). In Australia, State governments share the funding of public hospitals with the federal government. State governments provide the resources to the public hospital sector for outpatient and in-patient services, including use of telehealth services. While information on state government funding for this sector is not publicly available, respondents to the survey did report increases in the number of telehealth consultations within the public hospital sector.

Uncertainty about the future of government funding for Australian telehealth services after the pandemic dies down may exist because changes to Australian Government MBS funding rules over the course of the pandemic have proved difficult for some services, with one respondent complaining that “p*re COVID-19 was private billing and then 360 degree pivot to offer patients BB* [bulk billing] *and then 2 months later having to completely pivot again to a private billing only model*.” Other changes made to the original measures have meant that “*clients benefited from four months of Telehealth rebates (courtesy of COVID 19), only to have them wrenched away again*” which raises the question of whether equitable access to healthcare as advocated by the Australian Healthcare and Hospital Association (35) has been maintained during the pandemic.

Changes to the scale of and funding for Australian telehealth services were not the only indicator to change. Managerial, medical (and to a lesser extent technical) cultures were reported to have shifted to support the delivery of care via using telehealth services. Cultural changes have been previously identified as important to the implementation of health service changes in the Consolidated Framework For Implementation Research (CFIR) proposed by Damschroder et al. which “is composed of five major domains: intervention characteristics, outer setting, inner setting, characteristics of the individuals involved, and the process of implementation” (36). Damschroder et al. suggest that the CFIR provides “*a pragmatic organization of constructs upon which theories hypothesizing specific mechanisms of change and interactions can be developed and tested empirically*” (p. 3).

In the inner domain of the CFIR, culture constitutes the norms, values, and charter of an organization. Culture is an indicator of the readiness of an organisation and components of an organisation to undertake the work needed to bring about change (27). Respondents to our survey were of the opinion that organisational changes (in this case the extended coverage of telehealth services), were enforced changes, which were required to maintain the delivery of healthcare when healthcare professionals were physically separated from care recipients. One respondent summed up these changes:

*Managerial culture is much more supportive. Medical culture is much more supportive. Technical culture has always been supportive but has struggled to embed large volume telehealth services that are acceptable to both patients and staff*.

Our survey also measured other constructs posited within the CFIR, namely the need to build confidence in new practises and maintain supportive relationships. A respondent commenting on confidence in technologies stated that:

*Increasing confidence with video technologies - not just for telehealth - has led to increasing confidence in and use of technology to collaborate remotely - especially with an urgency to find ways to provide and sustain care for consumers*.

Teamwork, collaboration and networking amongst health professionals were identified as being very important, illustrated by this comment from a manager:

*our unit runs many meetings each week which have now been transformed to the use telehealth platforms. This has been extremely beneficial to keep things going on a service, education and patient review level. It has also allowed our regional colleagues to feel more like part of the service and partake*.

The CFIR (21) has been referred to in this discussion because it provides a contextually sensitive framework which groups constructs into outer (organisational or societal) settings and inner (professional) contexts. Pawson and Tilley (37) referred to the role of contexts in conditioning “the potential interactions between social or cultural structures and individual or collective agency” (p. 216). The corollary processes, where individual or collective agency expressed through social interactions influence contexts, are the mechanisms which influence social and cultural structures. Mechanisms may be layered and consist of one or more sub-mechanisms which can be considered analogous to the constructs posited by Damschroder et al. in the CFIR (21). Mechanisms operating in the social world “do” work: they can be seen as constructs, processes, or theories explaining “what it is about a program, in this case telehealth services, which makes it work.” Westhorp (24) has described mechanisms as processes with multiple inputs which interact with social actors to produce changes in social (and physical) contexts; that is, they are social interactions which have powers that produce change.

This study aimed to confirm, or otherwise, the influence of high-level social mechanisms that legitimise practise, build confidence in telehealth practises, support relationships between stakeholders, and acquire resources for the use of telehealth services to access healthcare during the COVID-19 pandemic. Analysis of responses to our survey shows that each of these proposed mechanisms have been able, in differing degrees, to “do work” to influence the changes to healthcare delivery resulting in a greatly increased volume and type of telehealth services. These mechanisms were largely triggered by changes in government regulations in response to the pandemic.

In turn, organisational and professional contexts have supported and adapted to the forced separation of care providers and patients during the pandemic. Organisational strategies and revised processes such as inclusion of telehealth consultations in patient appointment systems have supported the use of telehealth services. Professional cultures, especially managerial and clinical attitudes have shifted from hesitant support for remote consultations, to a determined encouragement of this modality. Respondents felt that telehealth services were now considered as routine care, and the Australian Minister of Health has said:

“We'll work now with all of the medical groups, just this evening I've spoken to the AMA [Australian Medical Association] and the college of GPs [about] using that period over the next six months to complete the process of consultations to make permanent that which we have already created on a temporary basis.”(38)

## Limitations and Strengths

The study has the following limitations. Table 1 shows that the majority of survey respondents were located in the Australian states of New South Wales, Queensland and Victoria. While these states have high levels of telehealth activity the conclusion we can draw in this paper may not be fully representative of all Australia states. While the number of respondents from these states provides a reasonable sample for the purposes of qualitative research, there remains the potential for bias in our quantitative assessment of the survey due to the limited number of respondents (*N* = 91).

Because participation in the survey was voluntary it is possible that only experienced providers of telehealth services elected to respond to the survey and the views of recent providers may not have been well represented. We did not ask participants if they were employed in public or private healthcare organisations, but it should be noted that funding of telehealth services, which is largely publically derived, whether or not the provider is a private operator. We have not commented on possible variations in adoption of telehealth services by state because the sub-sample sizes are too small to draws representative conclusions.

The survey was designed to ask close- and open-ended questions which sought to identify changes that had occurred in the contexts within which telehealth services operate, and to assess the mechanisms which had contributed to these changes. To our knowledge there has been no validated questionnaire developed specifically for assessing telemedicine adoption. Therefore, the survey questions were not designed to psychometrically measure the attitudes of respondents, which may reduce the validity of sections of our analysis.

In qualitative, research, the sample size required to provide adequate data to support research findings has been related to the point at which additional interviews provide no additional themes when the data is analysed. For mixed method, quantitative and qualitative surveys it is more difficult to define the number of survey responses that provide an adequate amount of data to support the findings (39). We have therefore indicated the number of responses that our discussion of draws on for the reader to make their own judgements as to the adequacy of the sample. While the limited sample sizes for the responses to some questions reduces the power of the conclusions that can be reached, one strengths of this survey is that it is the first survey in Australia to probe the social mechanisms that influence use of telehealth services to access healthcare and therefore lays the basis for further contextual sensitive research in this field.

## Conclusions

Organisational and professional contexts which contain social interactions are themselves not stable but evolve over time under the influence of mechanisms to form new states. Mechanisms that legitimise practise, build confidence in telehealth practises, support relationships between stakeholders, and acquire resources for the operation of telehealth services during the COVID-19 pandemic have been shown to interact with the organisational and professional contexts of Australian healthcare.

Triggered by the pandemic telehealth services have been legitimised to operate on a much larger scale than before and funding in Australia has supported this expansion. As a result of the need to physically distance care, acceptance and confidence in telehealth services as a modality of healthcare delivery has grown significantly. Looking forward to a period beyond the pandemic it is likely that there will be further changes to the regulatory regime for telehealth in Australia. How these changes will affect telehealth services remains to be seen but there have already been calls for health reform which would expand telehealth, encourage outreach and telehealth with new primary care models, better connect the public and private sectors, and expand out-of-hospital care (11).

The ongoing interaction between telehealth services, contexts and mechanisms is complex. The adoption of telehealth access to enable physically separated care, predominately using the telephone, may mark a “new context;” or it could be that once the pandemic passes, previous policies and practises will re-assert themselves and curb support for telehealth-enabled care.

## Data Availability Statement

The raw data supporting the conclusions of this article will be made available by the authors, without undue reservation.

## Ethics Statement

The studies involving human participants were reviewed and approved by Flinders University Social and Behavioural Research Ethics Committee. The patients/participants provided their written informed consent to participate in this study.

## Author Contributions

AT designed the survey instrument and wrote the first draught of the manuscript. HG analysed the quantitative data. AT and LC analysed the qualitative data. LC revised the manuscript. HG, PW, and AK critically reviewed and revised the manuscript. All authors contributed to the conception and design of the study, and read and approved the final manuscript.

## Conflict of Interest

The authors declare that the research was conducted in the absence of any commercial or financial relationships that could be construed as a potential conflict of interest.
